# A protein-dependent side-chain rotamer library

**DOI:** 10.1186/1471-2105-12-S14-S10

**Published:** 2011-12-14

**Authors:** Md Shariful Islam Bhuyan, Xin Gao

**Affiliations:** 1 Mathematical and Computer Sciences and Engineering Division, King Abdullah University of Science and Technology, Thuwal, 23955, KSA

## Abstract

**Background:**

Protein side-chain packing problem has remained one of the key open problems in bioinformatics. The three main components of protein side-chain prediction methods are a rotamer library, an energy function and a search algorithm. Rotamer libraries summarize the existing knowledge of the experimentally determined structures quantitatively. Depending on how much contextual information is encoded, there are backbone-independent rotamer libraries and backbone-dependent rotamer libraries. Backbone-independent libraries only encode sequential information, whereas backbone-dependent libraries encode both sequential and locally structural information. However, side-chain conformations are determined by spatially local information, rather than sequentially local information. Since in the side-chain prediction problem, the backbone structure is given, spatially local information should ideally be encoded into the rotamer libraries.

**Methods:**

In this paper, we propose a new type of backbone-dependent rotamer library, which encodes structural information of all the spatially neighboring residues. We call it protein-dependent rotamer libraries. Given any rotamer library and a protein backbone structure, we first model the protein structure as a Markov random field. Then the marginal distributions are estimated by the inference algorithms, without doing global optimization or search. The rotamers from the given library are then re-ranked and associated with the updated probabilities.

**Results:**

Experimental results demonstrate that the proposed protein-dependent libraries significantly outperform the widely used backbone-dependent libraries in terms of the side-chain prediction accuracy and the rotamer ranking ability. Furthermore, without global optimization/search, the side-chain prediction power of the protein-dependent library is still comparable to the global-search-based side-chain prediction methods.

## Background

Protein molecules are indispensable in most of the cellular functions, such as metabolism, gene regulation, signal transduction, and cell cycle. The capability of being such a diverse worker arises mainly due to their structures. Therefore, predicting protein structures accurately is important for both function determination and protein design purposes.

### Side-chain prediction

**Figure 1:**
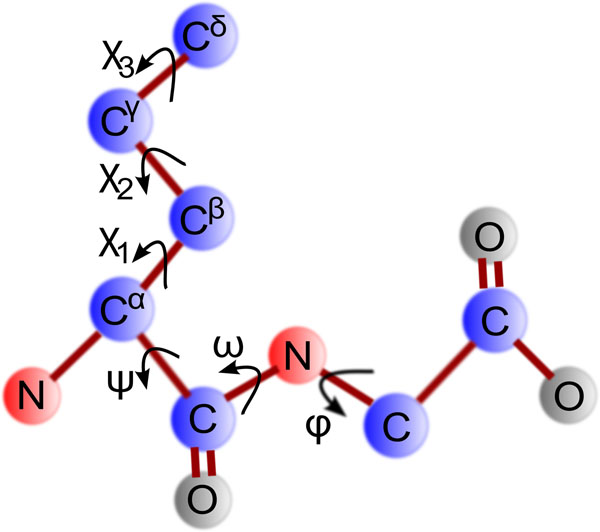
**Protein dihedral angle.** This figure illustrates different protein dihedral angles. *ϕ*, *ψ* and *ω* constitute backbone dihedral angles and *χ*_1_, *χ*_2_ and *χ*_3_ denote side-chain dihedral angles.

A protein structure contains both the backbone structure and the side-chain structure. Protein structures are typically represented in either coordinate space or angular space. Based on the assumption that the length of the covalent bonds is approximately constants, protein structures are usually modeled in angular space, which can reduce the number of variables by about one third. The dihedral angles can be calculated from coordinates that define the corresponding twists of the protein’s backbone as well as side chains. There are three backbone dihedral angles namely *ϕ*, *ψ* and *ω*. Each of them defines the twist between two corresponding neighboring planes defined by backbone atoms. To explain the detailed side-chain conformations, dihedral angles *χ* are used. Different amino acids have different number of side-chain dihedral angles with the maximum number of four, namely *χ*_1_, *χ*_2_, *χ*_3_ and *χ*_4_ respectively. Figure 1 illustrates the definition of the backbone and side-chain dihedral angles. The interesting thing is these angles cannot take any arbitrary values due to atomic clashes and orientations. They appear to take values only from discrete domains. These discrete conformations which are available to the side-chain dihedral angles are called **rotamers **[1].

Due to the difficulty of predicting complete protein structures simultaneously, structure determination remains as a multi-phase task. There are different sub-tasks including backbone prediction, side-chain prediction, loop modeling, and refinement. In this paper, we focus on the prediction of the side-chain conformation for a given backbone structure, i.e., protein side-chain prediction problem. By using the concept of rotamers, this is essentially the problem of correct rotamer assignment for every amino acid so that the overall structure is thermodynamically stable. It is assumed that stability comes at low internal energy states. That is why the problem of side-chain prediction is traditionally considered to be an optimization problem which strives to find a rotamer assignment which will minimize the total internal energy of the protein molecule. Since in most cases rotamers are discrete values, the problem is reduced to a combinatorial search problem in previous work [2-5].

To solve an optimization problem, two components are needed, the objective function which has to be maximized/minimized and the search strategy which tries to search for the global maximum/minimum. In side-chain prediction, the rotamer solution space is exponential in the size of the protein and the objective function, which is an energy function in this case, has numerous local minima. This combination dictates people to prioritize the candidate rotamers to design a practical search strategy, which is the place where rotamer libraries come to play a role. In the past three decades there have been lots of studies in each direction. Different kinds of energy functions have been tried and developed [4-10]. In the domain of search strategy, a broad range of combinatorial search algorithms, both exact [11-15] and approximate [16-20] ones, have been applied. To incorporate prior knowledge, different kinds of rotamer libraries have been developed. In this paper, we propose a novel idea in the context of rotamer library.

### Rotamer library

Rotamer libraries [16,21-24] are important components not only in side-chain prediction but also in several other areas including protein design. They summarize the existing knowledge of the experimentally determined structures quantitatively. Along with other information, rotamer libraries contain estimated probabilities of the discrete conformations of side-chain dihedral angles calculated from the structure databases. Depending on how much contextual information is taken into account, there can be different kinds of libraries. Initially the libraries consider only amino acid specific context and the probabilities are given for rotamers of different amino acids [25-30]. They are called backbone independent rotamer libraries. However, the discriminative power of the backbone independent libraries is not enough to eliminate sufficient amount of rotamer choices. Therefore, the backbone dependent rotamer libraries have been introduced [1,21-24,31-36]. These libraries consider the local backbone context through the *ϕ* and *ψ* angles along with the amino acid information. Backbone dependent rotamer libraries have been demonstrated to be able to boost the accuracy of a side-chain predictor equipped with the global optimizer over energy function landscape and guide them to avoid local minima [33]. Theoretically, the more context-specific information the library can encode, the more precise rotamer choices it can deliver. In this paper, we combine the general purpose backbone-dependent rotamer library with the detailed backbone atom coordinates of a specific protein, to introduce a **protein-dependent rotamer library**, without global optimization or search. To the best of our knowledge, this is a novel idea in the domain of rotamer library. For traditional backbone-dependent rotamer library, for a certain amino acid, the probability of its certain rotamer depends only on the local backbone *ϕ* and *ψ* angles. In our case the probabilities of two different rotamers of the same amino acid with the same *ϕ* and *ψ* angles can have different marginal distributions depending on their interactions with the surrounding environments.

### Markov random field model

Given a backbone dependent rotamer library, e.g., Dun-brack’s libraries published in the year of 2002 or 2010, and the backbone structure of a query protein, we first model the backbone and side-chain structures of the protein in Markov random field (MRF), where the residues are modeled as vertices of the interaction graph. We then employ widely used energy functions, e.g., Scwrl3 [4] energy function, to set up the potential for inference algorithms, e.g., sum-product belief propagation, to compute the marginal distributions of the residue-specific rotamers. In this way, all the rotamers are re-ranked for each residue in the query protein, according to the marginal distributions. We will demonstrate that this re-ranking can significantly improve the accuracy of the input backbone dependent rotamer library, which can hopefully benefit the global search algorithms for side-chain packing, such as the dead-end-elimination algorithm proposed in [11] and the tree decomposition algorithm proposed in [2,3].

One thing to notice is that modeling protein structures using probabilistic graphical models is not new [37-40]. Kamisetty et al. modeled protein structures by MRF and applied generalized belief propagation (GBP) to compute the free energy of a protein structure [37]. Our graphical model of protein structures is similar to their model. However, our focus is to calculate the marginal distributions and re-rank the rotamers, without calculating the free energy. We will demonstrate that loopy belief propagation (LBP) outperforms GBP for this purpose. Besides, we have encoded an energy function that is more suitable for re-ranking the rotamers than the ROSETTA energy function used in [37]. Yanover et al. modeled protein structures by conditional random field (CRF) and applied max-product belief propagation (BP) algorithms for side-chain prediction [39]. Our work is different from theirs in several ways. Firstly, their purpose is to apply max-product BP as a global search algorithm, which means they are interested in finding the optimal rotamer combination of all the side chains simultaneously, i.e., the combination that corresponds to the maximum joint probability. Therefore, their method is a side-chain predictor by itself, which can hardly be used by more powerful search algorithms, such as the one proposed recently in Scwrl4 [5]. We model protein structures as a MRF and apply sum-product BP, which provides the detailed marginal distribution for each side-chain, without global optimization. That means, if one selects the highest probability rotamer for each side-chain in our method, it may not yield a valid side-chain packing due to atomic clashes. Therefore, our method should be considered as a protein-dependent rotamer library which serves as the input for global search algorithms. Secondly, [39] used the ROSETTA energy function and demonstrated that the tree re-weighted BP algorithm performed very well to minimize and learn this energy function. However, our results demonstrate that this is not a general case. We use the simpler Scwrl3 [4] energy function and for that tree re-weighted BP does not perform better than the other BP algorithms.

Another thing to notice is that our protein-dependent rotamer library computes the marginal distributions of all the side-chain torsion angles (up to four) for a specific residue position, rather than considering them independently. This makes sense due to the high correlation between the torsion angles belong to the same amino acid.

### Contributions

Our contributions can be summarized as follows:

1. We introduce the idea of protein-dependent rotamer library and show the superiority of this library with respect to the widely used backbone-dependent rotamer libraries [1,24] in terms of both the accuracy of rotamer ranking and the probability assigned to the correct rotamers, on a large benchmark data set proposed recently by [5].

2. We model the protein structure as a MRF, encode the Scwrl3 energy function, and compare different sum-product BP algorithms to re-rank the rotamers. Our method does not contain a learning process, which is more likely to perform consistently well on other data sets and other energy functions.

3. The proposed protein-dependent rotamer library can be easily used as a side-chain predictor if we threshold each marginal distribution to its most probable rotamer. We compare our library with the most widely used side-chain predictors [2-5] and demonstrate that the accuracy is acceptable without using any global search/optimization techniques. Moreover, our library gives a probability distribution among rotamer choices instead of producing a single choice.

## Methods

We use the backbone structure of a protein in PDB format as our input. The output is a rotamer library with a format similar to that of Dunbrack’s library [1]. In Dunbrack’s library for each combination of an amino acid and a particular (*ϕ*, *ψ*) backbone dihedral conformation, there is one and only one distribution of rotamer conformations. However, in our generated output, for every amino acid in the protein sequence we have a distribution. This implies we can have distinct marginal distributions for same type of amino acids with similar (*ϕ*, *ψ*) angles. The distributions differ because of the consideration of surrounding environment of a certain amino acid.

When a protein backbone conformation is given, our method constructs an interaction graph where each residue is a vertex. We place an edge between a pair of residues if at least one pair of atoms from them is found to be closer than a minimum threshold. After that we set up the energy potentials for each node as well as each edge. Using the potentials, an inference algorithm is applied to calculate the marginal distributions of rotamers choices.

Our discussion of methods can be logically split into the following three phases:

1. Creating the interaction graph

2. Setting up energy potentials

3. Inferring marginal distributions

### Creating the interaction graph

**Figure 2:**
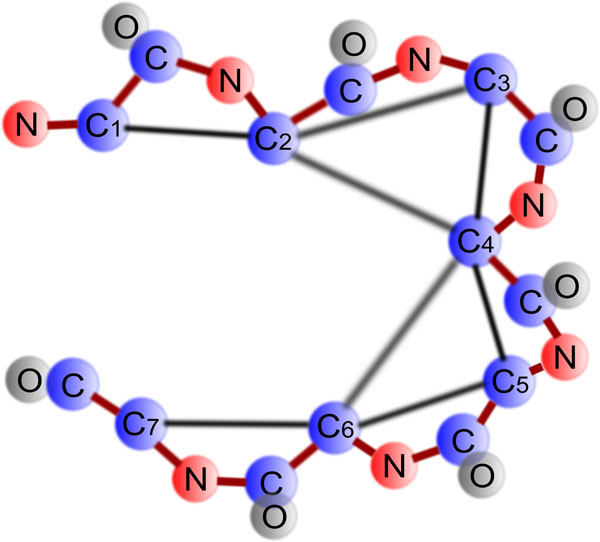
**Interaction graph for residue chain.** This figure gives an example of the interaction graph for a protein sequence of seven amino acids. *C_i_* denotes the alpha carbon of the *i*th residue. Apart from the neighboring relationships, the are two more edges in this map namely (*C*_2_, *C*_4_) and (*C*_4_, *C*_6_).

From the coordinates of the backbone atoms we create an interaction graph for the given protein. For every amino acid in the protein a vertex is added. We join each residue pair with an edge for which the distance between any possible pair of their corresponding *C^α^*, *C^β^* and carbonyl-oxygen atoms is less than the contact threshold. In our experiment, we set the contact threshold to 10Å which is the value used by ROSETTA. Figure 2 shows an illustration of an interaction graph for a protein sequence of seven amino acids. We denote the interaction graph as *G* = (*V*, *E*), where *V* is the set of all vertices and *E* is the set of all edges. For every vertex we calculate the backbone *ϕ* and *ψ* angles and load the corresponding amino-acid specific rotamer conformations from the input backbone-dependent rotamer library with the detail description of rotamers. The description contains possible discrete conformations along with their estimated prior probabilities, and the mean values of *χ*_1_, *χ*_2_, *χ*_3_, *χ*_4_, respectively.

### Setting potentials

**Figure 3:**
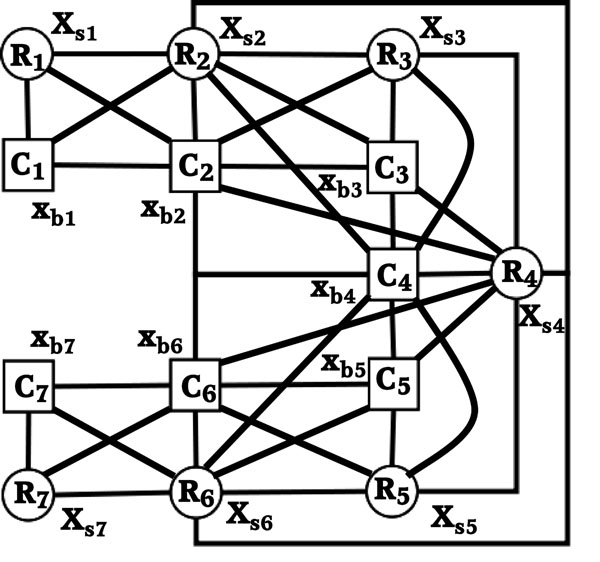
**Markov random field for interaction graph in Figure **2. This figure illustrates the corresponding Markov random field structure of the interaction graph from Figure 2. Square nodes are backbone random variables for which conformation is known and circular nodes are side-chain random variables for which conformation is unknown. *x_bi_* denotes the backbone conformation of the *i*th residue. Since this is given, the variable is not capitalized. *X_si_* denotes the side-chain conformation *R_i_* of the *i*th residue. Since this is not given, the variable is capitalized.

After creating the interaction graph, we calculate the energy potentials for the vertices and the edges. In MRF, potential functions are a measure of the likelihood for the random variables. We denote the entire protein structure by a set of random variables *X* = {*X_b_*, *X_s_*} where *X_b_* is our given backbone structure consists of *ϕ* and *ψ* angles and *X_s_* is the side chain structure consists of *χ*_1_, *χ*_2_, *χ*_3_, and *χ*_4_. We need to approximate the marginal probabilities for *X_s_*. Figure 3 shows the corresponding Markov random field model for the interaction graph shown in Figure 2. The probability for a specific assignment of rotamers is given by *P*(*X_s_* = *x_s_*|*X_b_* = *x_b_*, *θ*), where *x_s_* is a particular assignment of side-chain variables for a given backbone assignment *x_b_*, and *θ* is all the other parameters needed in prior to calculate the probabilities. Observations suggest that the side-chain conformation of a specific residue does not depend on every part of the protein. Therefore, it is assumed that only the residue pairs with an edge in the interaction graph can affect the side-chain conformation of each other. Using this conditional independence, for each vertex *V_i_* we can write the probability of a particular assignment by *p*(*X_i_* = *x_i_*|*Neighbors*(*X_i_*), *θ*). The probability of a complete side-chain assignment can be given by following equation:(1)

Here *C*(*G*) is the set of all cliques in *G*, *f* is a potential function denoting the likelihood of a specific assignment of the backbone and the side chain conformation, and *Z* is called a normalizing factor. Our potential function includes two components, i.e., vertex potential and edge potential. Vertex potential *E_i_* is contributed by the interactions between all the atoms of a certain residue *x_i_* and the backbone atoms of all the other neighboring residues. Edge potential *E*_*i*,*j*_ is contributed by the interactions among the side-chain atoms of a certain residue-residue pair (*x_i_*, *x_j_*) where *x_i_* and *x_j_* are connected by an edge in the interaction graph. To define potential function we use the Boltzmann distribution. According to the Boltzmann distribution the vertex potential for a node *x_i_* can be written as:(2)

Similarly the edge potential for a pair of vertices (*x_i_*, *x_j_*) can be written as:(3)

Here the *k_B_* is the Boltzmann constant and *T* is the absolute temperature. *k_B_T* = 0.6 kcal/mol is used. To define the vertex potential of a vertex *x_i_* for a particular rotameric state *r_ij_*, we use the following equation:(4)

Here *k_d_* is a constant factor, which is set to *k_d_* = 3.0. *p*(*r_ij_*) is the prior probability of the rotamer *r_ij_* and *p*(*r_i_max__*) is the probability of the most densely populated rotamer, both of which are for a specific backbone conformation of the vertex *x_i_* and are read from the input rotamer library. We use this component to prioritize more likely rotamers by giving them an energetically favorable advantage. Similar technique was used in Scwrl4 [5]. However, they used amino acid specific constants and learned them from training data. *E_sc_i__*(*r_ij_*) denotes the summation of all pairwise energy components resulting from the interaction between the atoms of residue *x*i and backbone atoms of all the other neighboring residues. To calculate the energy between two atoms *a* and *b* we use the energy function used by the SCWRL3.0 [4]. It is a piecewise function used to approximate the repulsive portion of the Lennard-Jones 12-6 potential. It can be defined as:

Here *d* is the Euclidean distance between the coordinates of the two atoms. If *r_a_* is the interaction radius of atom *a* and *r_b_* is the interaction radius of atom *b* then *r_ab_* = *r_a_* + *r_b_*. The default radius values used by Scwrl4 [5] are used here, i.e., *E_max_* = 10 and *k_sc_* = 0.8254. To define the edge potential between two vertices *x_i_* and *x_j_* for a particular rotameric pair (*r_im_*, *r_jn_*) we use the following equation:(5)

Here *E_scij_* (*r_im_*, *r_jn_*) denotes the summation of all pair-wise energy components resulting from the interaction between the side chain atoms of residue *x_i_* in rotameric state *r_im_* and the side chain atoms of residue *x_j_* in rotameric state *r_jn_*. For calculation of energy of an atomic pair we use the SCWRL3.0 function described above. The *E_hb_*(*x_i_*, *x_j_*) denotes the energy due to hydrogen bonding between residue pair. We use the hydrogen bond component of the ROSETTA energy function.

### Inferring marginal distributions

After assigning all the vertex and edge potentials, the interaction graph becomes a MRF. To re-rank the rotamer choices for each side-chain in this MRF, marginal distributions need to be computed. We employ different inference algorithms such as loopy belief propagation (LBP), generalized belief propagation (GBP) with a region graph, mean field approximation (MF) and tree re-weighted belief propagation (TRBP). Among them, LBP performs better than others, as we will show in the Results section. We give a brief description of them in the following.

#### Loopy belief propagation

In LBP, we initialize the vertices with some random marginal distributions called beliefs. In each iteration, depending on the potential function and the messages passed by the neighbors, every vertex updates its belief, which is assumed to be an approximation of the marginal distribution of rotamer choices for this vertex. After updating to new belief, the vertex forms a set of new messages for each of its neighbors and passes them accordingly. This procedure is repeated by every vertex at each iteration. For connected acyclic graphs it gives the exact marginal distributions for the random variables associated with the vertices of the graph. However, for the graphs with loops it gives a good estimate when the procedure converges. We set a maximum number of 100 iterations to detect whether it converges or not. If two successive iterations do not differ more than a threshold in their beliefs, the algorithm is considered to be converged. For scheduling we use an asynchronous update. The calculated belief or marginal distribution for a vertex *x_i_* in LBP is defined by the following equation:(6)

Message update rule is defined by the following equation:(7)

The first equation intuitively captures the marginal likelihood by combining old belief of a vertex and the old incoming messages sent by all of its neighbors. From this information a vertex can calculate new outgoing messages which capture an estimation of the marginal distribution of destination neighbors by combining old belief of the source vertex and the beliefs of source vertex estimated by all of its neighbors except the destination. Specifically, *b*(*x_i_*) denotes the approximated marginal distribution of the node *x_i_*. The set of all the neighbors of *x_i_* is represented by *N*(*i*). *m_ij_*(*x_j_*) indicates a message sent from node *x_i_* to node *x_j_* and contains a marginal distribution of node *x_j_* estimated by node *x_i_*. We use the sum-product algorithm for message passing where each node collects messages from all of its neighboring nodes and calculates new messages for each of its neighbors by taking the product of messages sent by other neighbors and summing over its current distribution.

#### Other inference algorithms

**Generalized belief propagation** is a family of approximate inference algorithms which divide the original graph into several regions to decrease the computational complexity. However, the belief expression and message update rule remain same with one subtle difference. Due to the division among regions one node can occur in multiple regions. So, we need to set weights for the contributions of these border nodes to different regions so that their overall contributions remain correct.

**Mean field approximation** tries to approximate the overall joint probability distribution by a product of independent marginals. This does not explicitly pass any messages, however at each iteration it tries to update its beliefs with the following equation:(8)

In **tree re-weighted belief propagation**, the regular loopy belief propagation is given another set of constants called edge appearance probabilities or *ρ_ij_* which represents the probability of an edge (*x_i_*, *x_j_*) that it will appear in a spanning tree of the graph. This is a mechanism for edge prioritization which affects the belief equation and the message update rule. The equation for belief can be written as the following equation:(9)

The message update rule can be written as the following equation:(10)

After computing the marginal distribution of side-chain conformation for every vertex, the rotamers in the input rotamer library are re-ranked for each side-chain. We create a protein-dependent rotamer library according to the same structure of the input backbone-dependent rotamer library which can be used by other global optimization algorithms.

### Dataset and software

To show the efficacy of our idea, we use the same data set of 379 proteins used in the Scwrl4 [5] paper. This is a larger and more recent data set comparing to the ones used in [2,4]. After downloading the PDB files, there are 355 of them which do not contain any duplicate backbone atoms. For this set we run our program and construct the protein-dependent rotamer library, then compare the performance with two other widely used backbone-dependent rotamer libraries [1,24]. For calculating actual dihedral angles from the original PDB files, we use the program Dangle [41]. To create the interaction graph of the protein we use the molecular biology toolkit [42]. Please note that since our method does not involve any training process, we do not subdivide the data set.

## Results

In this section, we evaluate the performance of our proposed protein-dependent rotamer library. First of all, we compare the side-chain packing power of our library and the widely used backbone-dependent libraries [1,24]. For our protein-dependent library, we threshold the marginal distribution of each side-chain to its most probably rotamer, which is considered as the prediction of our library for side-chain packing purpose. For the backbone-dependent libraries, we select the rotamer with the highest probability as the prediction. Please note that neither our library nor the backbone-dependent libraries involve global optimization. However, strong side-chain packing power gives more potential for the global optimization/search algorithms to benefit from the library. Secondly, we evaluate the re-ranking accuracy of our protein-dependent rotamer library. We compare both the average rank of the first correct rotamers and the average probability of finding correct rotamers within the top 1, 2 and 3 rotamers, respectively. A lower average rank and a higher probability can clearly reduce the search space of the following global optimization/search algorithms and boost the likelihood of such algorithms to pack side-chains correctly. Finally, we compare the accuracy and the speed of the four inference algorithms.

To calculate the accuracy of a rotamer choice, the most widely used criterion is used, i.e., if the mean dihedral angle of this rotamer is within 40 degree of the actual dihedral angle, this rotamer is considered to be correct; otherwise, it is considered to be wrong. For *χ*_1+2_ to be correct, both *χ*_1_ and *χ*_2_ have to be correct. We judge the correctness of *χ*_1+2+3_ and *χ*_1+2+3+4_ similarly.

### Performance on side-chain prediction

We first evaluate the side-chain packing power of our protein-dependent rotamer library. We choose the widely used backbone-dependent rotamer libraries proposed by Dunbrack’s lab in 2002 and 2010 [1,24] for comparison. The backbone-dependent library is used as input for our method and the corresponding rotamers are re-ranked according to the marginal distributions. For both our protein-dependent libraries and the backbone-dependent libraries, the rotamer with the highest probability for each side-chain is considered as the prediction by the corresponding library. The predictions are then compared with the real side-chain angles to calculate the accuracy.

In this experiment, we use LBP as the inference algorithm, because as we will show later in this section, LBP outperforms the other three inference algorithms. Similar conclusion can be drawn if other inference algorithms are used.

Table 1 shows the performance of four rotamer libraries namely

**Table 1:** Comparison of rotamer libraries for side-chain prediction

Amino acid	Dihedral angle	P10	P02	D10	D02
**CYS**	*χ*_1_	55.76	**56.40**	50.16	50.09

**SER**	*χ*_1_	**67.34**	67.13	61.94	61.84

**THR**	*χ*_1_	**88.46**	87.81	86.13	85.85

**VAL**	*χ*_1_	**90.79**	90.58	86.94	86.99

**ASN**	*χ*_1_ *χ*_1+2_	**79.21** **56.18**	78.33 53.34	69.53 49.34	69.31 47.15

**ASP**	*χ*_1_ *χ*_1+2_	78.27 **60.80**	**79.33** 60.36	72.47 57.16	73.12 56.18

**HIS**	*χ*_1_ *χ*_1+2_	**79.12** **45.01**	77.93 43.29	63.33 33.33	62.06 32.86

**ILE**	*χ*_1_ *χ*_1+2_	**91.56** **77.71**	91.18 77.20	86.91 68.18	87.05 68.02

**LEU**	*χ*_1_ *χ*_1+2_	**84.21** **74.22**	83.70 73.19	74.89 68.59	74.20 67.94

**PHE**	*χ*_1_ *χ*_1+2_	**88.26** **53.17**	86.90 52.28	72.95 42.00	73.03 42.23

**PRO**	*χ*_1_ *χ*_1+2_	**83.11** **79.01**	82.20 78.19	80.96 76.70	80.92 76.74

**TRP**	*χ*_1_ *χ*_1+2_	**69.42** 55.60	68.06 50.40	53.49 35.61	53.01 34.98

**TYR**	*χ*_1_ *χ*_1+2_	**87.29** 51.30	86.38 **51.59**	72.64 42.67	72.68 43.18

**GLN**	*χ*_1_ *χ*_1+2_ *χ*_1+2+3_	**72.37** **50.25** **25.71**	70.67 48.91 23.08	63.62 34.05 17.20	62.47 38.72 16.03

**GLU**	*χ*_1_ *χ*_1+2_ *χ*_1+2+3_	**67.46** **47.86** **26.17**	66.39 46.65 25.03	62.36 41.97 21.21	61.71 41.14 20.48

**MET**	*χ*_1_ *χ*_1+2_ *χ*_1+2+3_	71.54 56.50 **39.95**	**72.40** **56.66** 39.51	60.03 36.31 20.12	60.85 34.55 19.91

**ARG**	*χ*_1_ *χ*_1+2_ *χ*_1+2+3_ *χ*_1+2+3+4_	**71.52** **56.83** **29.92** **17.60**	71.35 56.60 29.62 16.82	63.60 47.18 21.33 9.82	61.41 47.47 21.27 9.14

**LYS**	*χ*_1_ *χ*_1+2_ *χ*_1+2+3_ *χ*_1+2+3+4_	72.02 58.54 **44.83** **28.42**	**72.11** **58.79** 44.80 27.85	66.43 50.82 36.86 23.33	66.28 50.73 36.89 23.48

**Overall**	*χ*_1_ *χ*_1+2_ *χ*_1+2+3_ *χ*_1+2+3+4_	**80.45** **61.50** **32.81** **23.25**	80.05 60.74 31.82 22.55	73.80 53.72 24.62 16.94	73.43 53.60 24.03 16.61

The first column contains amino acid names. The second column denotes the combination of dihedral angles for which the accuracy is reported. Starting from the third column, the accuracy for our proposed library with Dunbrack’s 2010 library as input, our proposed library with Dunbrack’s 2002 library as input, Dunbrack’s backbone-dependent library proposed in 2010, and Dunbrack’s backbone-dependent library proposed in 2002 is reported, respectively.

**D02** Dunbrack’s backbone-dependent rotamer library proposed in 2002 [1]

**D10** Improved version of Dunbrack’s library proposed in 2010 [24]

**P02** Our protein-dependent rotamer library with D02 as the input library

**P10** Our protein-dependent rotamer library with D10 as the input library

The accuracy of *χ*_1_ until *χ*_4_ (if there exists) for different amino acids as well as the overall accuracy of the four rotamer libraries is shown in Table 1. It can be seen that our protein-dependent library clearly outperforms both D10 and D02 on all the amino acids. In fact, the *χ*_1_ accuracy of P10 improves the higher one of D10 and D02 by at least 5% on 15 out of all the 18 amino acids, whereas the improvement is at least 10% on five amino acids. The overall *χ*_1_ accuracy of both P10 and P02 is above 80%, which improves the corresponding input library by about 6.5%. We also run a well-known side-chain prediction method, TreePack, proposed in [2], which is based on a global search algorithm, i.e., tree decomposition, on the same data set. The overall accuracy of TreePack is about 82%. This demonstrates that without global optimization/search, our protein-dependent rotamer library is still comparable to the global search methods.

One thing to notice is that the improvement of the accuracy of our libraries is not consistent on different amino acids. There are some amino acids whose accuracy has been improved significantly (around 15-20%). There are also few amino acids whose improvement is below average. We investigate the fact and discover that accuracy of all the amino acids with a big aromatic ring has been improved greatly. They are HIS, PHE, TRP and TYR. A possible explanation is that because of the size of the aromatic rings, the conformations of the amino acids with aromatic rings highly depend on the local geometric environments, rather than depending only on backbone information. These amino acids are more constrained in choosing a particular rotamer even if the rotamer is heavily represented within the database. Therefore, *ϕ* and *ψ* angles, which are the only information used by backbone-dependent rotamer libraries, are not enough to reveal the conformation preference of such side-chains. Therefore, the simple statistics from the generic protein databases can be misleading.

One interesting thing is that on MET, which does not have any big aromatic ring, our protein-dependent rotamer libraries still have about 10% improvement on *χ*_1_ and about 20% improvements on *χ*_1+2_ and *χ*_1+2+3_. It turns out that MET is the only amino acid which has a sulfur atom inside its side chain (not the end of the side-chain). Sulfur has a bigger atomic radius with respect to carbon and nitrogen. So the conformation of sulfur dihedral angles are more constrained than the carbon or nitrogen dihedral angles, thus largely depend on the specific protein structure. However, this explanation can be questioned because of the low improvement of the accuracy for CYS, which also has a sulfur atom in its side-chain. This is due to the fact that in proteins, if suitable condition found, two CYS amino acids normally form a disulfide bond which changes its regular conformation. Such trend can already be partially captured by the statistics on the protein databases. Therefore, the protein-dependent rotamer libraries do not encode much more information than the backbone-dependent rotamer libraries. On the other hand, the energy function used in our method does not contain a specific term for disulfide bond, whereas side-chain prediction programs, which apply global search techniques, normally encode such a term. Therefore, it can be expected that our method does not improve the backbone-dependent libraries on CYS as much as the global search methods do.

It is shown in Table 1 that the overall accuracy of D10 is slightly higher than D02. Consequently, the overall accuracy of P10 is also higher than P02, which demonstrates that the improvement of our method is consistent and not input library dependent. Therefore, with an improved backbone-dependent or backbone-independent rotamer library, a better overall accuracy can be expected for our method.

### Performance on rotamer ranking

To demonstrate the potential for the global optimization/search algorithms to benefit from our protein-dependent rotamer library, we further evaluate the ability to re-rank the input rotamers of our library. It has been shown in Table 1 that P10 is better than P02 and D10 is better than D02. Therefore, from now on, we will use only P10 and D10 for comparison.

We first evaluate the average rank of the first correct rotamers for P10 and D10. The average rank of correct rotamers is calculated by taking the mean rank of the first correct rotamer for each side-chain by the corresponding library according to their probability. This indicates the expected rank within which a correct rotamer should be found. In ideal case, the average rank should be 1, which means the rotamer library is able to rank the correct rotamers as the first choice. The comparison of the average rank between P10 and D10 is shown in Table 2. It can be seen that P10 is able to improve the average rank of the first correct rotamers from 1.74 to 1.63 for *χ*_1_ and from 2.95 to 2.67 for *χ*_1+2_. This improvement denotes that our method re-ranks the original input rotamer library in such a way that the correct rotamers bubble up across the list. This is an important measurement since most of the global search procedures give a priority towards highly probable rotamers from the library. Usually such prior knowledge is encoded in the energy functions of the side-chain prediction methods. The result confirms that our library indeed prioritizes correct rotamers on average.

**Table 2:** Comparison of rotamer libraries for rotamer ranking

Average rank of correct rotamer		
**Dihedral angle**	**Rank of P10**	**Rank of D10**

*χ*_1_	**1.6301**	1.738
*χ*_1+2_	**2.6663**	2.9517

Average probability of finding correct rotamers at top 1 position		

**Dihedral angle**	**Probability of P10**	**Probability of D10**

*χ*_1_	**0.8018**	0.6470
*χ*_1+2_	**0.6111**	0.4127

Average probability of finding correct rotamer at top 2 positions		

**Dihedral angle**	**Probability of P10**	**Probability of D10**

*χ*_1_	**0.8984**	0.8899
*χ*_1+2_	**0.7248**	0.7053

Average probability of finding correct rotamer at top 3 positions		

**Dihedral angle**	**Probability of P10**	**Probability of D10**

*χ*_1_	**0.9313**	0.9265
*χ*_1+2_	**0.7655**	0.7479

The top part of the table shows the comparison of the average rank of the first correct rotamers between our protein-dependent rotamer library with Dunbrack’s 2010 library as input (P10) and Dunbrack’s backbone-dependent library proposed in 2010 (D10). The other part of the table shows the comparison of the average probability of finding correct rotamers in the top 1, 2 and 3 rotamers of P10 and D10, respectively.

Another set of criteria which has been evaluated is whether our top choices are populated by correct rotamers or not. We calculate the average probability of finding correct rotamers in the top 1,2 and 3 choices. As shown in Table 2, if we only consider the first choice, the average probability of correct rotamers boosts up from 0.65 to 0.80 for *χ*_1_ and from 0.41 to 0.61 for *χ*_1+2_. In the cases of top 2 and top 3 choices, even though the probability of both libraries are high, our library still outperforms the backbone-dependent library. Note that the probability here is the prior probability by the corresponding library, which is different from the accuracy of the library. Such prior probabilities are widely used in the energy functions of the global search algorithms to direct the search procedure. Therefore, with high average probability, the energy functions can be more accurate, which can thus reduce the search space of the side-chain packing methods.

Combining the results from Table 1 and Table 2, our protein-dependent rotamer library significantly increases the average accuracy for side-chain prediction, reduces the average rank of the first rotamers, and assigns higher prior probabilities to correct rotamers. All of these improvements are done without doing global optimization/search, which clearly shows the potential of the protein-dependent library to benefit the side-chain packing methods.

### Comparison of inference algorithms

We finally report the comparison between different inference algorithms for MRF on our problem. We compare the performances of four approximate inference algorithms namely,

**LBP** Loopy belief propagation

**GBP** Generalized belief propagation

**MF** Mean field approximation

**TRBP** Tree re-weighted belief propagation

Table 3 shows the average accuracy for side-chain prediction, average rank of the first correct rotamers, and the average running time of these four inference algorithms. With the exception of TRBP, all the other three algorithms perform similarly while LBP maintains a consistent superiority. This may look contradicting to the results reported in [39], in which they applied TRBP to optimize the ROSETTA energy function for side-chain prediction. However, in [39], they were interested in finding the side-chain configuration of the maximum probability and optimizing the energy function. Therefore, they applied max-product TRBP. Here, we are not interested in finding a final configuration. Instead, we want to estimate the marginal distribution of each side-chain accurately. Therefore, selecting the highest probability choice for each side-chain according to our library may not give a side-chain packing of the entire protein without atomic clash. Sum-product TRBP turns out to perform poorly for this purpose. There are two possible reasons for the weak performance of TRBP. Firstly, we use a different energy function than [39]. Instead of using ROSETTA we use SCWRL energy function. Secondly, TRBP is not well-suited for the sum-product algorithm which we use. This can be further backed up by the fact that while LBP fails to converge for only 3% of all the input proteins, TRBP fails to converge on more that 20% input proteins.

**Table 3:** Comparison of inference algorithms

Comparison				
**Attribute**	**LBP**	**GBP**	**MF**	**TRBP**

Accuracy of *χ*_1_	**80.07**	80.03	79.54	76.45
Accuracy of *χ*_1+2_	**60.78**	60.72	60.33	55.58
Average rank of *χ*_1_	**1.50**	1.51	1.54	1.59
Average rank of *χ*_1+2_	**2.23**	2.25	2.33	2.45
Average execution time (in seconds)	29.99	63.19	**14.17**	189.94

Comparison of the average accuracy for side-chain prediction, the average rank of the first correct rotamers, and the average running time for loopy belief propagation (LBP), generalized belief propagation (GBP), mean field approximation (MF), and tree re-weighted belief propagation (TRBP).

## Discussion

We have demonstrated that by modeling protein structures by MRF and applying inference algorithms to estimate the marginal distributions of the side-chains, we can get a much more accurate rotamer library, which we refer to as protein-dependent rotamer library. One may argue that although we do not use the global optimization/search algorithms, our method encodes the energy information. However, the energy information we used is mainly for the purpose of setting the potentials to build MRF, rather than for directing any search procedure. In this sense, the traditional backbone-dependent rotamer libraries also encode the energy information, in another form. The traditional libraries are mainly based on the statistics of the solved protein structures, which are assumed to be the global minimum conformation of the natural energy function. Therefore, doing statistics on such structures also encode energy information. This is further confirmed by the facts that if high-resolution protein structures are used to build the traditional libraries or if the core regions with high electri-density are used to do the statistics, the accuracy of the traditional libraries can be increased significantly [5]. Therefore, although our library uses energy functions in a more explicit way than the traditional libraries, it can be expected that the global search algorithms can still benefit a lot from our library. Since the source code for both SCWRL and TreePack are not publicly available, we can not directly encode our library into such global search methods. We are implementing our own dead-end-elimination, tree-decomposition and other search algorithms, such as A* search, to test the performance of combining our library with global search algorithms.

Although probabilistic graphical models are a relatively new tool for protein structure modeling, they have already proved their efficacy. However, they are not immune from all kinds of drawbacks. In our use of belief propagation, it is not guaranteed that the inference algorithm will converge. We avoid this problem by setting maximum limit on the number of iterations. Nevertheless, for our dataset loopy belief propagation is able to converge within 100 iterations for around 97% input proteins. Moreover, for those cases where LBP fails to converge, we still have moderately good results. Thus, this limitation is not as much daunting as it first seems to be. Other deterministic methods also can suffer from errant input. For example, both TreePack [2,3] and Scwrl4 [5] use tree decomposition technique to employ exhaustive search strategy. However, there is still no guarantee that the tree width of the tree decomposition must be small. Therefore, it is also possible that for some large input proteins, such methods may also fail to produce results or find an approximate solution.

One important application of our method is side-chain prediction for flexible backbone conformations. In many applications, a large number of backbone structures are available, such as the protein structure sampling, protein structures gathered from different protein structure prediction servers, or protein backbone refinement tasks. In such cases, there are a large number of close-to-native backbone structures, but none of them is the native structure. The traditional side-chain packing methods usually take only one single backbone structure as input, which cannot be applied here, because the set of structures contain important information about the native structure. Therefore, all of these close-to-native structures should be considered simultaneously. Our method can easily take a set of flexible backbone structures as input. In this case, the backbone structures will also be modeled as random variables. The standard belief propagation algorithms can still be used to infer the marginal distributions for side-chain rotamers under the condition of flexible backbones.

## Conclusion

In this paper, we have proposed a novel type of backbone-dependent rotamer library, i.e., protein-dependent rotamer library, which encodes structural information of all the spatially neighboring residues. By estimating the marginal distributions of the side-chains in a Markov random field model, the proposed library significantly boosts the accuracy of the input rotamer library, without global optimization or search. The proposed library can hopefully lead to the performance improvements of the side-chain prediction methods.

## Competing interests

The authors declare that they have no competing interests.

## Authors' contributions

XG initiated the idea. MSIB and XG wrote and revised the manuscript. MSIB carried out the experiments.
